# Liposomal Drug Delivery Systems and Anticancer Drugs

**DOI:** 10.3390/molecules23040907

**Published:** 2018-04-14

**Authors:** Temidayo O. B. Olusanya, Rita Rushdi Haj Ahmad, Daniel M. Ibegbu, James R. Smith, Amal Ali Elkordy

**Affiliations:** 1 School of Pharmacy and Pharmaceutical Sciences, University of Sunderland, Sunderland SR1 3SD, UK; Temidayo.olusanya@sunderland.ac.uk (T.O.B.O.); ritahajahmad@yahoo.co.uk (R.R.H.A.); 2 Department of Medical Biochemistry, College of Medicine, University of Nigeria Enugu Campus, Nigeria; daniel.ibegbu@unn.edu.ng; 3 School of Pharmacy and Biomedical Sciences, University of Portsmouth, Portsmouth PO1 2DT, UK; james.smith@port.ac.uk

**Keywords:** liposomes, anticancer drugs, drug delivery

## Abstract

Cancer is a life-threatening disease contributing to ~3.4 million deaths worldwide. There are various causes of cancer, such as smoking, being overweight or obese, intake of processed meat, radiation, family history, stress, environmental factors, and chance. The first-line treatment of cancer is the surgical removal of solid tumours, radiation therapy, and chemotherapy. The systemic administration of the free drug is considered to be the main clinical failure of chemotherapy in cancer treatment, as limited drug concentration reaches the tumour site. Most of the active pharmaceutical ingredients (APIs) used in chemotherapy are highly cytotoxic to both cancer and normal cells. Accordingly, targeting the tumour vasculatures is essential for tumour treatment. In this context, encapsulation of anti-cancer drugs within the liposomal system offers secure platforms for the targeted delivery of anti-cancer drugs for the treatment of cancer. This, in turn, can be helpful for reducing the cytotoxic side effects of anti-cancer drugs on normal cells. This short-review focuses on the use of liposomes in anti-cancer drug delivery.

## 1. Introduction

There is a high demand for advanced delivery systems that are suitable for the delivery of various active pharmaceutical ingredients (APIs), especially systems with low costs, high efficiency, low risks, and toxicity [1]. Several APIs can be utilised better by employing nano-size drug delivery systems (DDS) that are designed to enhance the delivery of APIs with poor pharmacokinetics and biodistribution [2]. For instance, most of the chemotherapeutic medications are characterised by poor pharmacokinetic profiles in addition to non-specific distribution in the body tissues and organs, causing serious side effects and systemic toxicity [3]. Accordingly, nano-size structures-based pharmaceutical formulations (e.g., liposomes [4], polymeric nanoparticles [5], electrosprayed particles [6], and nanosuspension [7]) have demonstrated better therapy of the APIs. Moreover, due to the complexity of solid tumours, an effective penetration of anti-cancer agents encapsulated within a nanocarrier is the main challenge in cancer therapy [8]. Liposomes are the most commonly investigated nanostructures used in advanced drug delivery, which were first discovered by Alee Bangham in 1963 [9]. Liposomes are artificially spherical vesicles prepared from naturally-derived phospholipid. They entail one or more lipid bilayers with discrete aqueous spaces. They are well established for a range of pharmaceutical and biomedical applications with the unique capability of entrapment of both hydrophilic (polar) and hydrophobic (nonpolar) compounds due to their amphipathic nature in aqueous media. For instance, hydrophobic compounds entrap in the bilayer membrane, while hydrophilic compounds encapsulate in the aqueous core [10]. Liposomes serve as DDSs due to their versatile structure; biocompatibility; and the fact they are naturally nontoxic, non-immunogenic, and biodegradable [11]. Liposomes have several advantages contributing to drug delivery. They have a role enhancing drug solubility [12], serving as a sustained release system [13], providing targeted drug delivery [14], reducing the toxic effect of drugs [15], providing protection against drug degradation [16], enhancing circulation half-life of APIs [17], being effective in overcoming multidrug resistance [18], improving the therapeutic index of the entrapped drug [19], and protecting APIs against their surrounding environment [20].

## 2. Cancer

Cancer is a life-threatening illness that leads to irregular and uncontainable growth of malignant cells. These uncontrollable cells can invade normal tissues and organs, causing undesirable growth and reactions that end up destroying them [21]. Cancer is responsible for ~3.4 million deaths worldwide [22]. Some of the well-known causes for cancer disease are smoking (causing lung [23], breast [24], and ovarian cancer [25]), being overweight or obese (associated with 13 types of cancer disease, such as breast cancer, kidney, womb and bowel cancers), intake of processed meat [26], radiation (causes skin cancer) [27], family history, stress, environmental factors, and chance [28].

Cancer cells are able to spread around the human body through blood vessels and lymphatic streams, causing metastasis by forming a secondary tumour [29]. Anticancer agents are typically administered to the patients to kill cancer cells. These drugs work in two ways: by killing the cancer cells through direct exposure to the chemical agent and by inducing apoptosis (suicide of cancer cells) [30].

Tumour vasculature is vital to preserving the tumour and aiding its growth [31]. It is characterised by special physiological properties, being highly chaotic, complex, and porous in nature. Pore size ranges between 100–780 nm, while normal tissue junctions are <6 nm [32]. The distance between tumour cells should be within a certain limit of the perfused blood vessels in order to obtain the required quantity of oxygen and nutrients to survive and proliferate [31]. Accordingly, tumours can undergo extensive angiogenesis (the growth of new tumour blood vessels), grow beyond 1–2 mm in diameter [31], and form hypervasculatures, which can be defective and impair the lymphatic drainage systems. In addition to these properties, tumours can produce three known vascular-permeability influences such as bradykinin, nitric oxide [33,34,35], and peroxynitrite [36]. Targeting the tumour vasculature is essential for tumour treatment. This can be done by disturbing the angiogenesis using antiangiogenic agents (eg, Axitinib (Inlyta®), Bevacizumab (Avastin®) and Cabozantinib (Cometriq®)) or shutting down the existing tumour blood flow, resulting in ischemia and tumour cell necrosis using vascular targeting agents (eg, 5,6-dimethylxanthenone-4-acetic acid (flavonoid derivative) and Combretastatin A-4 phosphate (tubulin binding drug) [37]. Accordingly, there is a high demand for finding a proper controlled anticancer dosage to control this disease, as it is expected to be responsible for 13.2 million death cases by 2030 [29].

Liposomal drug formulations offer the possibility of increasing efficacy while reducing the toxic side effects of chemotherapeutic drugs. They can also impact the pharmacokinetics and tissue distribution of the incorporated anticancer compound. They have been described as alternative DDSs that have been used to enhance the therapeutic index and significantly reduce the toxic effect of anticancer agents on normal tissues, including doxorubicin.

## 3. Categories of Liposomes

Numerous factors define liposomes properties such as the lipid composition, number of lipid bilayers, size, surface charge, and the method of preparation [11]. Liposomal vesicles vary in size between 0.025 µm to 2.5 µm. They can be categorised according to the number of their layers (also referred to as lamellae): unilamellar (consisting of single phospholipid bilayer) or multilamellar (consisting of more than one unilamellar separated by layers of water [38] (>500 nm)). Unilamellar vesicles are subdivided into small unilamellar vesicles (20–100 nm) and large unilamellar vesicles (>100 nm). Both the size and the number of lamellae in the liposomal structure are considered to be the most crucial factors affecting the vesicles half-life and the quantity of API that is to be encapsulated [11]. This unique and flexible variety in the liposomal structure distinguishes liposomes as the preferred carriers for a broad spectrum of therapeutic agents.

## 4. Stability of Liposomes

Physical and chemical stability of the liposomes in terms of size distribution, entrapment efficiency, and minimal degradation of liposomal apparatuses is the major limiting step for drug delivery using this system. Chemical degradation of liposomes mainly occurs at the phospholipid bilayers level, in which two different reactions might develop: (i) hydrolysis of the ester bonds between fatty acids and glycerol backbone, and (ii) peroxidation of any available unsaturated acyl chain. These two reactions might lead to the development of short-chain lipids; subsequently, soluble derivatives will appear in the membrane that would significantly reduce the quality and stability of the liposomal system [39]. With respect to physical instability, liposomes might undergo aggregation/flocculation and fusion/coalescence, which can ultimately change vesicle size and lead to significant loss of the encapsulated API [40].

Aqueous dispersions of liposomes suffer from elevated levels of instability due to the leakage of the encapsulated drug out from the phospholipid bilayers. In addition to this, aggregation of liposomes upon storage for a period exceeding the first few months of preparation was reported [41]. Accordingly, it is more advisable to store liposomal preparations in solid form. Several methods are available that show extendable techniques to stabilise the liposomal formulations, such as lyophilization [42], spray drying [43], and supercritical fluid [44].

Several factors that have an influence on liposomal system stability, such as liposomal composition (e.g., phospholipids-lipids with high phase transition temperatures), fatty acid side-chains, polar head chemistry, chain length, and the degree of unsaturation, are preferred to maintain liposomal rigidity [45] and phospholipid:cholesterol molar ratio (crucial for the liposomal stability and controlling drug release). Briuglia et al. (2015) demonstrated that 70:30 molar ratio of phospholipids (using 1,2-Dimyristoyl-sn-glycero-3-phosphocholine (DMPC), dipalmitoyl phosphatidylcholine (DPPC), and distearoyl phosphatidylcholine (DSPC)): cholesterol achieved a liposomal formulation that can guarantee the stability and control over drug release [40] and surface potential (high surface potential is directly related to the liposomal physical stability, as it helps to reduce the rate of fusion and aggregation [46].

One of the reasons for liposomal aggregation is the vesicle–vesicle electrostatic effect between the vesicles. Repulsive interactions, which are at least equal to the degree and range of the van der Waals force, are an essential requirement for stable liposomal formulation [39]. The physical stability of liposomes improves by increasing the surface charge density and reducing the ionic strength of liposomes (increases the electrostatic repulsive energies), especially when phosphatidylcholine and phosphatidylserine are used [46,47]. However, scientists need to consider all influencing factors affecting liposomal stability and work around all of them. This is due to the fact these factors might be affected by certain factors that can disrupt the system. For instance, electrostatic stabilisation is very sensitive to the surface charge (pH) and salt concentration of the liposomal suspension. Electrostatic stabilisation can be improved by combining it with the steric stabilisation (so called electrosteric stabilisation), which can be obtained by covering the surface of the liposomes with an adsorbed coat of long, bulky molecules (which, for example, keep the distance between the vesicles) [39].

## 5. Influence of Liposomal Composition in Drug Delivery

Phospholipids are the main building blocks of liposomes. These biomolecules are also the main components building the biological membranes. They are amphiphilic molecules that consist of a polar head (water soluble hydroxy groups) and insoluble backbone. Liposomes can be zwitterionic, positively or negatively charged, or uncharged. This is totally dependent on the polar head charge. There are two types of lipids currently utilised for liposome preparation: naturally occurring or synthesised double-chain lipids (consisting of phosphorus polar head and glycerol backbone) and sterols (e.g., cholesterol) [48].

The most known lipids used in the liposomal formulations are phosphatidylcholine (zwitterionic), phosphatidylglycerol (negatively charged), phosphatidic acid, phosphatidylethanolamine (zwitterionic), and phosphatidylserine (negatively charged). Positively charged lipids (e.g., *N*-[1-(2,3-dioleyloxy)propyl]-*N*,*N*,*N*-triethylammonium (DOTMA) and 1,2-dioleoyl-3-trimethylammoniopropane (DOTAP)) are mainly used for gene delivery, as they interact with the negatively charged deoxyribonucleic acid (DNA) [49] and negatively charged APIs.

Cholesterol is another strategic component of liposomes. It has a modulatory effect on the properties of the lipid bilayer of the liposomes. It can control the stoutness in the liposome structure [40] and increase the packing between the phospholipid molecules [50], resulting in more ordered conformation in the aliphatic tail region, reduced micropolarity [51], reduced bilayer flexibility to the surrounding molecules (especially water soluble molecules [52]), and increases in the microviscosity of the bilayer [53]. Cholesterol is also crucial for structural stability of liposomal membranes against intestinal environmental stress [51]. Cholesterol was found to influence liposomes size (increasing cholesterol concentration increases liposomes size in addition to shape transition), provide permeability and fluidity, and consequently modulate the release of hydrophilic compounds from liposomes [54].

**Figure 1:**
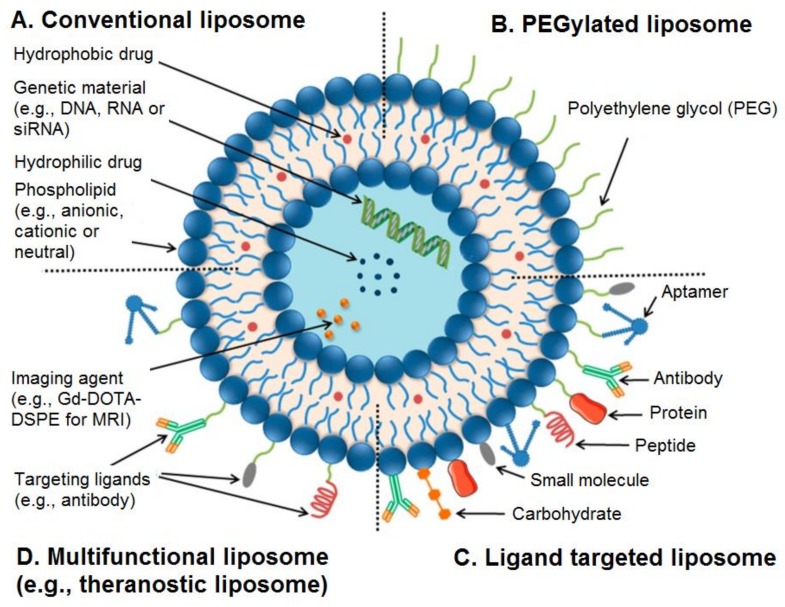
Structure of conventional and functionalised liposomes: (**A**) conventional liposomes comprising phospholipids; (**B**) PEGylated/stealth liposomes containing a layer of polyethylene glycol (PEG); (**C**) targeted liposomes containing a specific ligand to target a cancer site; and (**D**) multifunctional liposomes, which can be used for diagnosis and treatment of solid tumours. Adapted from Creative Commons Attribution License [55].

It is also possible to use surface functionalisation of liposomes by a variety of agents to overcome the limitations of these nanocarriers in terms of biological and physiological barriers [55]. For example, liposomes can be functionalised with polyethylene glycols (PEGs), aptamers, antibodies, proteins, peptides, ligands, carbohydrates, or small molecules (Figure 1) [55].

## 6. Liposomes as Targeted Drug Delivery System for Cancer Treatment

Advancements in liposomal vesicle development have achieved both controlled drug release and targeted drug delivery (disease-specific localisation). This property is essentially helpful for cancer treatment as surgical resection, radiation therapy, and chemotherapy are the first-line treatment of cancer. Some cancerous states require systemic administration of the chemotherapy. So far, most of the APIs used in chemotherapy have been highly cytotoxic to cancer and normal cells. Therefore, they suffer from plenty of side effects and limitations, as the free drug is administrated directly into the blood stream that circulates the body. The chemotherapeutic agent can then be uptaken by cancer and normal tissues, leading to severe toxicity to different body organs, such as heart, kidneys, liver, and others. As a result, sometimes the highest possible dose of chemotherapy is administrated to the patients to maximise the quantity of the medication taken up by the cancer cells [56]. The success of cancer treatment basically depends on its capability to decrease the size and remove tumours without affecting normal tissues, thus increasing patients’ survival time and enhancing their quality of life [29].

**Figure 2:**
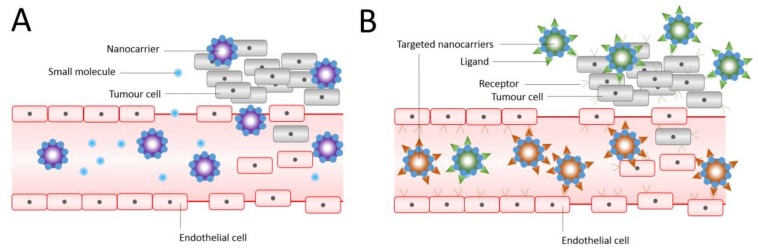
Passive (**A**) and (**B)** active targeting of nanocarriers. Nanocarriers reach tumours selectively through the leaky vasculature, or in other cases, where the nanocarrier size determines the retention in the tumour tissue. Drugs in the absence of nanocarriers diffuse freely in and out the tumour blood vessels due to their small size, and therefore their effective concentrations in the tumour decrease rapidly. The EPR effect is where drug-loaded nanocarriers cannot diffuse back into the blood stream due to their large size, resulting in progressive accumulation. In active targeting, ligands grafted at the surface of nanocarriers bind to receptors (over)expressed by cancer cells or to angiogenic endothelial cells. Adapted and reproduced with permission [3].

The encapsulation of chemotherapeutic agents within liposomal structures can limit the normal tissue uptake of the medication and thus improve its therapeutic index. By means of passive targeting, liposomes can concentrate preferentially on the tumour (typically over 24–48 h) via the enhanced permeability and retention (EPR) effect of the vasculature, in which leaky tumour vessels unite with absent lymph drainage [57]. In other words, passive targeting of liposomes happens by transferring them into the tumour interstitium via leaky tumour vasculature through molecular movement within fluids (Figure 2) [29]. Liposomes can actively target tumour tissues using the antibody-based approach. This can be done by adding certain antibodies to the liposomal surface—so called immunoliposomes (ILP)—which are specific to the cancer cells or to the endothelial cells of the tumour vasculature [58]. Maruyama et al. (1999) developed the pendant type ILP (34A-PEG-ILP), which is a long-circulating polyethylene glycol (PEG)-ILP attached to antibodies (34A antibody) at the distal end of PEG chain. These ILPs showed high targetability to the site of action (lung endothelial cells and tumour tissue)—more than ordinary liposomes. This is mainly caused by the effect of free PEG, which successfully helped to avoid the RES uptake of the ILPs [59]. The limitation of this approach is that not all tumour tissues or cells have a specific antigen for the targeted antibody to bind to. Accordingly, this approach is limited to the antigen-antibody specifications. 

An additional targeting method has been developed that uses an external trigger to solve this problem. This can be done by triggering the release of the chemotherapeutic agent within the interstitium after accumulating on the tumour tissue. This can be achieved by the effect of EPR or by releasing the agent within the tumour vasculature using liposomes particularly designed to respond to a precise external trigger (e.g., heat) [56,58]. For instance, thermosensitive liposomes that can be administered systemically were developed.

There are several strategies by which local hyperthermia could improve the effectiveness of the liposomal formulation for drug delivery: by inducing drug release at a temperature close to that of the lipid phase transition of the liposomes, by promoting blood supply to the site of action, by improving liposomes accumulation at the site of action by increasing endothelial permeability to liposomes, by enhancing the permeability of the target cells to API that release from the liposomes, by enhancing the fusion or endocytosis effect of target cells to the directly transferred drug from liposomes, and by improving drug release from liposomes by reducing the local pH of the target site of action [60]. The first thermosensitive liposomes were developed by Yatvin et al. (1978), in which the formulation used the two lipids DPPC:DSPC (molar ratio of 3:1), encapsulating neomycin (an aminoglycoside antibiotic) as API. A local hyperthermia is then initiated (>40–42 °C), triggering drug release at the targeted site [61]. The first animal test of an anticancer formulation using thermosensitive liposomes was tested by Weinstein et al. in 1979. A mouse with lung cancer was treated with methotrexate-encapsulated thermosensitive liposomes (DPPC:DSPC, 7:3 molar ratio). The results showed a 4-fold increase of the drug quantity that reached the tumour tissue. However, the elimination of the liposomes within 1 hour of administration was the major limitation of this formulation [60].

It was suggested by Magin et al. (1986) that the clearance and distribution of temperature-sensitive liposomes is size-dependent. The size range of 50–200 nm was recommended [61], as the endothelium tissues in the kidney glomerulus have a pore size of 40–60 nm. However, macrophages in liver and spleen can easily remove these liposomes from blood circulation, as the pore size of sinusoidal endothelium in liver and spleen is around 150 nm [29]. More studies demonstrated that vesicles size, lipid composition, surface coating and charge [62], and liposome-plasma protein interaction all have an impact on the clearance pharmacokinetics of liposomes by the reticuloendothelial system [63]. Therefore, the selection of the most appropriate lipids (e.g., lysolipid temperature-sensitive liposomes) [64], incorporation of cholesterol (to increase vesicles stability and reduce drug leakage) [65], and the use of optimum polymers for coating [66] can help improve this DDS. For instance, coating PEG onto liposomes is a helpful approach that can prevent liposome engulfment by the macrophages and thus increase their blood circulation time. Maintaining an optimum hyperthermal effect is another major limitation in clinical settings using thermosensitive liposomes, as it can result in overheating of tissues. Magnetic liposomes-mediated unit chemotherapy and hyperthermal effect was developed as a potential solution to overcome this problem. For instance, a novel magnetic liposomal formulation for self-controlled hyperthermia and chemotherapy was designed by Gogoi et al. (2017). Liposomes co-encapsulated with dextran-coated biphasic suspension of La_0.75_Sr_0.25_MnO_3_ (LSMO) and iron oxide nanoparticles were developed using paclitaxel (PCX) as a model drug. Evaluation of the therapeutic efficacy of the formulation showed a 2.5-fold (mean tumour volume 2356 ± 550 mm^3^) reduction of the tumour growth after a single administration of the drug-loaded magnetic liposomes. A 3.6-fold (mean tumour volume 1045 ± 440 mm^3^) reduction of the tumour growth after a double dose treatment was reported as compared to the growth reduction effect of the corresponding control (mean tumour volume 3782 ± 515 mm^3^). With no significant leaching of liposomes, biocompatibility and therapeutic evaluation studies demonstrated the potential use of magnetic liposomal formulation for the treatment of drug-resistant or physiologically vulnerable cancer [67]. The combination of chemotherapy and thermotherapy was also reported using doxorubincin (DOX)-loaded magnetic liposomes (using citric acid-coated magnetic nanoparticles). About 130 nm-sized magnetic liposomes were developed utilising hydrogenated soy phosphatidylcholine (HSPC)/1,2-distearyl-sn-glycero-3-phosphoethanolamine (DSPE)/cholesterol (12.5:1:8.25 molar ratio) and DOX by rotary evaporation and ultrasonication process. In vitro cytotoxicity and hyperthermia studies evaluated against colorectal cancer revealed that the magnetic liposomes displayed no cytotoxicity, with approximately 56% tumour cells being killed. This study demonstrates the effectiveness of the combination of hyperthermia and chemotherapy treatment in one system as compared with individual treatment [68].

Another approach for the delivery of anticancer drugs is using enzyme-responsive liposomes. The idea for this approach came after detecting high concentrations of certain enzymes in patients diagnosed with cancer. For instance, some extracellular enzymes, e.g., secreted phospholipase A2 (sPLA_2_) (raises in prostate [69], breast [70] and pancreatic [71] cancers), matrix metalloproteinases (MMPs) (specifically, MMP-2 and MMP-9 elevates in breast [72], colorectal [73], pancreatic [74], and lung [75] tumours), urokinase plasminogen activator (uPA) (elevated in a number of human cancers, such as breast, colon, bladder, and ovarian tumours) [76], elastase (found in high concentration in cases of lung [77], breast [78], and skin [79] tumours), prostate-specific antigen (PSA) (raises in case of prostate tumour) [80], and some intracellular enzymes, e.g., cathepsin B (elevated in brain, breast, prostate, and lung cancer) [81,82].

PEGylated, sterically stabilised liposomes can stabilise the entrapped API, enhance activity, change API disposition, and reduce toxicity [83]. However, they are unable to control their drug release kinetics [84]. Over expression of the over-mentioned enzymes could be an effective target for controlling drug release from liposomes [85]. This was reported for DOX-loaded sPLA2-responsive liposomes developed by Mock et al. (2014) for the treatment of prostate cancer. Liposomes were prepared with cholesterol, DSPC, DSPE, and (1,2-distearoyl-sn-glycero-3-phosphoethanolamine-*N*-[poly(ethylene glycol) 2000)] (DSPE-PEG2000) (10 µmol total phospholipid). Animal tests showed that the liposomes were 1.5 to 2 times more effective than sterically stabilised liposomes (composed of DSPC, cholesterol, and DSPE-PEG2000) at reducing tumour growth. The mechanisms mediating enzyme and drug uptake, toxicity, and disposition of liposomes are cell- and formulation-dependent [86].

Cancer immunotherapy by vaccination is not yet a major type of treatment for cancer. However, many scientists are searching for new approaches and formulations that could be effective in the use of immunotherapy for cancer treatment or prevention. Allison and Gregoriadis were the first to report the capability of liposomes to induce immune responses of entrapped antigens [87,88]. Several types of cancer vaccines currently under investigation such as tumour cell vaccines, antigen vaccines, dendritic cell vaccines, and vector-based vaccines. Depending on the chemical properties of materials used in cancer vaccination, hydrophilic antigens (e.g., nucleic acids, peptides, carbohydrates, proteins, and haptens) encapsulate within the aqueous phase of liposomes, whereas hydrophobic compounds (e.g., antigens, linker molecules, lipopeptides, and adjuvants) are intercalated into the outer lipid bilayer. Antigens and adjuvants, which are also lipophilic molecules, can be adsorbed or chemically linked to the liposome surface [89]. For instance, a combination of Rituximab® and non-PEGylated liposomal DOX are recommended as front-line therapy for the treatment of elderly patients (80 years old) against aggressive non-Hodgkin lymphoma [90].

## 7. Applications of Liposomes in Anticancer Drug Formulations

Chemotherapeutics are the first line treatment approach for cancer. However, most of them are limited due to unconcealed toxicity, poor selectivity of the right tissues, narrow therapeutic index, and high probability of developing drug resistance. These factors can lead to dramatic failure in cancer treatment. The development of nanoscale liposomal formulations has been shown to help the selective transportation of the drug to the tumour cells [91]. This consequently avoids the off-target toxicity due to the EPR effect [92]. Some liposomal-based DDS were approved by the FDA with potent anticancer activity. Table 1 summarises some of the liposomal formulations used as cancer treatments.

DOX is an anthracycline antibiotic (isolated from *Streptomyces peucetius* var. *caesius*) with potent anticancer activity that is widely used in the treatment of solid and hematologic neoplasms (such as breast cancer and lymphoma) [93]. However, its clinical uses are limited by considerable cardiotoxicity and cytotoxicity. The cardiotoxicity is usually cumulative and mostly leads to irreversible damage to the cardiomyopathy and congestive heart failure, which is the result of generation of free radicals and lipid peroxidation [93,94]. DOX can affect the DNA in several ways: (I) the cytotoxicity effect of DOX originates mostly from its intercalation that occurs at the DNA double helix minor groove level through electrostatic interactions of sugar moieties with phosphate residues; (II) DOX has the potential to stabilise DNA-topoisomerase II in a way that could prevent the DNA double helix resealing and thus stop the cell replication process; and (III) by apoptosis, which can also be triggered by the DNA break repair process [95].

Abbreviations: DPPC, dipalmitoyl phosphatidylcholine; DSPC, distearoyl phosphatidylcholine; PCX, paclitaxel; LSMO, La_0.75_Sr_0.25_MnO_3_; DOX, doxorubincin; HSPC, hydrogenated soy phosphatidylcholine; DSPE, 1,2-distearyl-sn-glycero-3-phosphoethanolamine; DSPE-PEG2000, 1,2-distearoyl-sn-glycero-3-phosphoethanolamine-*N*-[poly(ethylene glycol) 2000]; DNR, daunorubicin: ATRA, all-*trans* retinoic acid; DOTAP, 1,2-dioleoyl-3-trimethylammoniopropane; MXT, mitoxantrone; DSPE2000_,_ 1,2-distearoyl-sn-glycero-3-phosphoethanolamine-methoxy PEG2000; TPGS1000-TPP, d-α-tocopheryl polyethylene glycol 1000 succinate-triphenylphosphine conjugate.

Doxil®, a PEGylated liposomal-based DOX (100 nm with 10,000–15,000 DOX per liposome), was the first nano-drug to be approved by the FDA in 1995 for the treatment of AIDS-related Kaposi’s sarcoma, in 1999 for the treatment of ovarian cancer, and in 2007 for the treatment of multiple myeloma. The formulation is composed of hydrogenated soy phosphatidylcholine, cholesterol, and a lipid with a PEG head group (DSPE-PEG2000) in 56.4:38.3:5.3 mole ratio [101]. It shows a potent anticancer activity based on the following principles: (i) due to the use of PEGylated liposomes that result in stealth formation (in which segments of methoxypolyethylene glycol (hydrophilic) are attached onto the surface of each liposome (which covers 60% the liposomal surface)), this enables the drug to last longer in the blood stream (3–4 days in humans), avoiding the RES and improving the extravasation through endothelial gaps in tumours [102]; (ii) the “liquid ordered” of the lipidic bilayer in the liposomes that consist of phosphatidylcholine (high melting point 53 °C) and cholesterol; and (iii) efficient, high, and stable loading of doxorubicin in the liposomal formulation through the effective use of the transmembrane ammonium sulfate gradient, allowing drug release at the tumour site [103]. The main challenge for this formulation lies in having the maximum drug loading efficiency during the manufacturing steps. PEGylated DOX hydrochloride liposome (PEGADRIA) was developed by Ali et al. (2016) with a similar physiochemical profile to Doxil®. The only difference between the formulations is that in PEGADRIA, DOX hydrochloride was used. PEGADRIA was found to be safe and effective, as well as Doxil®, in the treatment of patients with ovarian cancer [104]. It is believed that PEGylation of the liposomal formulation Doxil® is the reason behind its toxicity. Myocet® is another liposomal-based, DOX citrate-encapsulated formulation (180 nm and composed of 1-palmitoyl-2-oleoylphosphatidylcholine:cholesterol, mole ratio 55.8:44.2), but it lacks the PEGylated coating. It is characterised by a shorter circulation time compared with Doxil®, with dramatically reduced cardiac toxicity [15,96].

Daunorubicin (DNR) is another anthracycline antibiotic (isolated from *Streptomyces peucetius varcaesitue*) drug with anticancer activity [97]. It works the same as DOX with significant side effects, such as cardiotoxicity (dose-dependent), alopecia, nausea, and vomiting, which are associated with DNR therapy, but liposomal daunorubicin formulation has been developed as an alternative to reduce some of these adverse side effects. Liposomal DNR (DaunoXome®), 50 nm in size and composed of DSPC:cholesterol in a molar ratio of 2:1, was the only first-line therapy for the treatment of advanced HIV-associated Kaposi’s sarcoma [105], approved by the FDA in 1996. It was designed to be phagocytosed by monocytes in the blood circulation. Its small size and the fact it is uncharged help to minimise RES uptake and consequently to prolong drug circulation time [97]. The most prominent side effect of this formulation is severe leukopenia [106] and neutropenia in some cases [107]. The use of this formulation in treatment has shown higher levels of plasma DNR in treated mice compared to the free drug. Likewise, the clearance of the free drug was shown to be 44.9 mL/h, while the liposomal formulation was 0.195 mL/h, demonstrating a very slow clearance rate of the DaunoXome®. In another related study, it was reported that DaunoXome® displayed a favorable cardiac toxicity profile, also allowing an increased anthracycline dose without increasing cardiotoxicity [108]. Treating patients with Kaposi sarcoma using liposomal DNR formulation at a dose of up to 1700 mg/m^2^ showed no cardiotoxicity [105].

**Figure 3:**
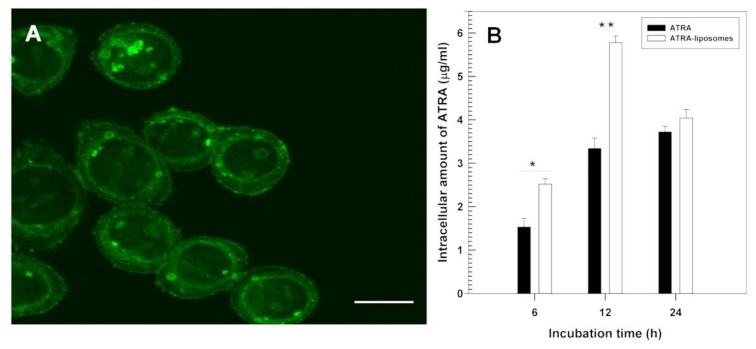
(**A**) Confocal laser scanning micrograph showing the interaction between fluorescein-labeled liposomes and FRO cells after 6 h incubation (bar = 35 μm) and (**B**) intracellular uptake of ATRA as free form or entrapped in liposomes within FRO cells as a function of the incubation time. Reproduced with permission [16]. * *p* < 0.05, ** *p* < 0.01.

All-*trans* retinoic acid (ATRA) is an anti-tumour agent characterised by different anti-cancer activities against several types of cancer cells [16]. Due to its common side effects (burning of the skin and general malaise) after systemic administration, having a highly variable degree of bioavailability, and its propensity to induce its own metabolic degradation after oral administration, an alternative pharmaceutical formulation is required. Negatively-surface charged ATRA-loaded liposomes (∼200 nm) made up of DPPC/cholesterol/1,2-distearoyl-sn-glycero-3-phosphoethanolamine-methoxy PEG2000 (6:3:1 molar ratio) were developed and investigated against three different human thyroid carcinoma cell lines (PTC-1, B-CPAP and FRO) (Figure 3). These liposomes were found to protect ATRA against photodegradation and enhance its antiproliferative properties, promoting them to be a novel formulation for the treatment of anaplastic thyroid carcinoma [16]. ATRA was also encapsulated within cationic liposomes aiming for the treatment of lung cancer. In the formulation composed of DOTAP, cholesterol, and ATRA (70:20:10 molar ratio), liposomes (263 nm) contained ca. 92% of entrapped drug, and animal tests showed significant high bioavailability in lung and blood. As compared with free drug, a higher half-life of 14.8 vs. 13.2 h), C_max_ (0.66 vs. 0.29 μg/mL), and a lower clearance (46.6 vs. 136.3 μg/mL/h) in vivo was reported for the liposomal formulation demonstrating the potential for lung cancer treatment [98].

Mitoxantrone (MXT) (1,4-dihydroxy-5,8-bis(((2-[(2-hydroxyethyl)amino]ethyl)amino))-9,10-anthra-cenedione dihydrochloride) is an anthracenedione antineoplastic drug. It is commonly utilised for the treatment of several types of cancer such as lymphomas, breast cancer, prostate cancers, and leukemias [109]. It has been proposed that it works by numerous mechanisms of action: intercalation, calcium release, electrostatic interactions with DNA, inhibition of topoisomerase II, DNA-protein cross-links, immunosuppressive activities, and inhibition of prostaglandin biosynthesis by interfering with the regulatory process, which is dependent on the hydroperoxy fatty acid [110]. Animal studies suggested that free MXT has also cardiotoxic potential. However, the drug leads to less severe cardiac damage than free DOX, as it acts using a different mechanism [111]. MXT liposomes intended for the treatment of lymphoma and breast cancer are still in phase II trials. An animal study was conducted by Wang et al. (2010) to compare the pharmacodynamics, pharmacokinetics, and tissue distribution of MXT loaded-liposomal and free MXT. It was found that from the pharmacodynamic studies, that MXT loaded-liposomes inhibited tumour growth (at a dose of 1 to 4 mg kg^-1^); from the antitumour effect studies that MXT loaded-liposomes enhanced the therapeutic effect of the drug significantly in comparison with free MXT; from the pharmacokinetic studies that MXT loaded-liposomes had longer circulation characteristics as compared with free MXT at the same dose; and from tissue distribution studies that MXT loaded-liposomes accumulated into tumour zones rather than normal tissues. The overall studies suggested that the therapeutic effect and MXT therapeutic index could be improved by using MXT loaded-liposomes [19]. Another liposomal formulation developed by Legut et al. (2014) suggested the addition of anacardic acid improved the encapsulation and retention of MXT. A liposomal formulation composed of HSPC: DSPE-PEG2000: cholesterol: anacardic acid in molar ratio of 0.55:0.05:0.35:0.05 was stable for 6 months, with over 90% encapsulation efficiency in 5 min. The formulation showed anticancer activity towards melanoma cell lines. The cytotoxicity of MXT was also improved [99].

**Figure 4:**
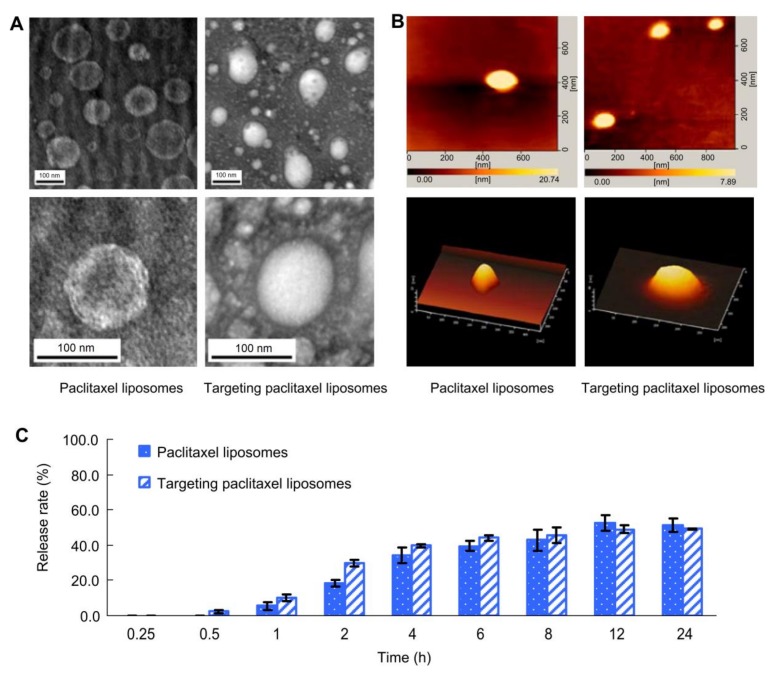
Imaging and release rates of paclitaxel (PCX) liposomes: (**A**) transmission electron microscopy images of PCX liposomes and targeting PCX liposomes, (**B**) atomic force microscopy images of PCX liposomes and targeting PCX liposomes, and (**C**) release rates (%) of PCX-loaded liposomes in the release media of pH 7.4 PBS containing 10% fetal bovine serum (mean ± standard deviation (n = 3). Reproduced with permission [30].

PCX is a naturally occurring compound extracted from *Taxus brevifolia* [30]. PCX has an anticancer activity against several cancers including refractory ovarian, breast, non-small cell lung cancer, gastric cancer [112], head and neck tumours, urologic malignancies, and Kaposi’s sarcoma [113]. This alkaloid compound works by a unique mechanism of action. During the mitotic phase of cell division, PCX promotes polymerisation of cancer cells tubulin proteins, disturbing their dynamic, stabilising their microtubules, making them dysfunctional, and, finally, leading to cell death. However, PCX anticancer activity is limited due to its highly lipophilicity (log P value of 3.96) and very poor water solubility (<0.01 mg/mL) [113]. Taxol® is the pharmaceutical formulation of PCX administrated in a non-aqueous base vehicle. It has caused severe hypersensitivity reaction to patients as a result of Cremophor EL in the formulation in addition to the precipitation on aqueous dilution [114]. Accordingly, the encapsulation of this active agent can be helpful for targeting the cancer cells. PCX loaded liposomes are designed to target and accumulate at the mitochondria in cancer cells using d-α-tocopheryl polyethylene glycol 1000 succinate-triphenylphosphine conjugate (TPGS1000-TPP) as a mitochondrial targeting compound. The liposomal formulation is composed of egg phosphatidylcholine, cholesterol, and TPGS1000-TPP in molar ratio of 88:3.5:8.5, respectively. Administration of this formulation (>85% encapsulation efficiency) showed the strongest inhibitory effect on tumour cells’ growth at day 26 (73.14% of tumour volume inhibitory rate). The small particle size (80 nm) of liposomes in addition time and to TPGS1000-TPP conjugate at the liposome membrane both played a role in enhancing the circulation escape of the rapid elimination by RES. Moreover, PCX liposomes triggered apoptosis of drug-resistant lung cancer by stimulating the pro-apoptotic Bax and Bid proteins and suppressing the anti-apoptotic Bcl-2 protein (Figure 4) [30]. A comparison study was conducted to evaluate the efficacy and safety of PCX liposome (Lipusu®) and free PCX on patients suffering from gastric cancer. Patients received other chemotherapeutic agents (tegafur and oxaliplatin usually described for patients with advanced gastric cancer) at the same time in the study. Both formulations showed almost the same hematological and neurological toxicities (*P* > 0.05), while significant reductions on PCX side effects (e.g., nausea, hypersensitivity reactions, and vomiting) were reported for patients receiving the PCX liposomal formulation [112].

Wang-Gallam et al. (2018) report the findings of a global, phase 3, randomised, open-label trial at 76 sites in 14 countries with patients with metastatic pancreatic ductal adenocarcinoma [100]. This study (NAPOLI-1) assessed the effect of a nanoliposomal formulation of irinotecan, alone and in combination with fluorouracil (FU) and folinic acid (FA). The nanoliposomal irinotecan formulated with the FU and FA combination extended the survival of patients with the disease whom had previously received gemcitabine-based therapy, thereby opening a new treatment option.

In summary, liposomes offer an attractive vehicle for targeted delivery of anticancer agents. These nanocarriers overcome the undesirable side effects that range from simple headache to cytotoxicity in normal tissues and increase circulation time, bioavailability, and target-site drug concentration accumulation; they also reduce elimination and toxicity, and protect the chemotherapeutic agents from the surrounding environment, in addition to carrying them to the desired site of action. Liposomes may be surface-functionalised using a range of biomolecules to achieve the required receptor-specific targeting [115].

## Figures and Tables

**Table 1:** Liposomal formulations used as anticancer treatments.

Active Ingredient	Liposome Composition	Size (nm)	Cancer Type Being Targeted		Reference
DOX	HSPC/DSPE/cholesterol (12.5:1:8.25 molar ratio)	130	Colorectal (in-vitro)		[68]
DOX	Cholesterol, DSPC, DSPE and DSPE-PEG2000 (10 µmol total phospholipid).	100	Prostate cancer (in-vivo/in-vitro		[86]
DOX	HSPC: cholesterol: lipid with a PEG head group (DSPE-PEG2000) (molar ratio 56.4:38.3:5.3)	100	Colorectal (in-vitro)		[68]
DOX	1-Palmitoyl-2-oleoylphosphatidylcholine: cholesterol (molar ratio 55.8:44.2)	180	Metastatic (clinical trial & in clinic)		[15,96]
DNR	DSPC:cholesterol (molar ratio 2:1)	50	Kaposi’s sarcoma		[97]
ATRA	DPPC:cholesterol:1,2-distearoyl-sn-glycero-3-phosphoethanolamine - Methoxy PEG2000 (molar ratio 6:3:1)	200	Human Thyroid carcinoma (in-vitro)		[16]
ATRA	DOTAP, cholesterol and ATRA (molar ratio 70:20:10)	263	Lung cancer (in-vivo in animal)		[98]
MXT	HSPC: DSPE-PEG2000: cholesterol: anacardic acid (molar ratio 0.55:0.05:0.35:0.05)	112	Melanoma cell lines (in-vitro)		[99]
PCX	Egg phosphatidylcholine: cholesterol: TPGS1000-TPP (molar ratio 88:3.5:8.5)	80	Lung cancer cell lines (in-vivo & in-vitro)		[30]
Irinotecan	-	-	Pancreatic ductal adenocarcinoma		[100]
